# Improved functional expression of recombinant human ether-a-go-go (hERG) K^+ ^channels by cultivation at reduced temperature

**DOI:** 10.1186/1472-6750-7-93

**Published:** 2007-12-20

**Authors:** Mao Xiang Chen, Shaun L Sandow, Virginie Doceul, Yu Hua Chen, Heather Harper, Bruce Hamilton, Helen J Meadows, Derek J Trezise, Jeff J Clare

**Affiliations:** 1 BR&AD, GlaxoSmithKline R&D, Stevenage, UK; 2 Department of Physiology and Pharmacology, University of New South Wales, Sydney, Australia; 3 BR&AD, GlaxoSmithKline R&D, Harlow, UK

## Abstract

**Background:**

HERG potassium channel blockade is the major cause for drug-induced long QT syndrome, which sometimes cause cardiac disrhythmias and sudden death. There is a strong interest in the pharmaceutical industry to develop high quality medium to high-throughput assays for detecting compounds with potential cardiac liability at the earliest stages of drug development. Cultivation of cells at lower temperature has been used to improve the folding and membrane localization of trafficking defective hERG mutant proteins. The objective of this study was to investigate the effect of lower temperature maintenance on wild type hERG expression and assay performance.

**Results:**

Wild type hERG was stably expressed in CHO-K1 cells, with the majority of channel protein being located in the cytoplasm, but relatively little on the cell surface. Expression at both locations was increased several-fold by cultivation at lower growth temperatures. Intracellular hERG protein levels were highest at 27°C and this correlated with maximal ^3^H-dofetilide binding activity. In contrast, the expression of functionally active cell surface-associated hERG measured by patch clamp electrophysiology was optimal at 30°C. The majority of the cytoplasmic hERG protein was associated with the membranes of cytoplasmic vesicles, which markedly increased in quantity and size at lower temperatures or in the presence of the Ca^2+^-ATPase inhibitor, thapsigargin. Incubation with the endocytic trafficking blocker, nocodazole, led to an increase in hERG activity at 37°C, but not at 30°C.

**Conclusion:**

Our results are consistent with the concept that maintenance of cells at reduced temperature can be used to boost the functional expression of difficult-to-express membrane proteins and improve the quality of assays for medium to high-throughput compound screening. In addition, these results shed some light on the trafficking of hERG protein under these growth conditions.

## Background

Human ether-a-go-go related gene (hERG, KCNH2) potassium channels mediate the rapid activating delayed rectifier potassium current (I_Kr_) in ventricular myocytes. Loss of function mutations in hERG or the administration of channel blockers cause a delay in membrane potential repolarization of the cardiac action potential, and give rise to a prolongation of the QT interval on the electrocardiograph ([1], for review). This 'long QT syndrome' can trigger polymorphic ventricular dysrhythmias (*torsades de pointes*), syncope and sudden death.

The hERG channel is blocked by a plethora of structurally diverse compounds, including many marketed drugs, some of which, such as terfenadine and cisapride, have been withdrawn from the market due to this unwanted activity [1-3]. For this reason there is strong interest within the pharmaceutical industry in optimizing the recombinant expression of hERG, in order to improve the quality of medium to high-throughput assays for detecting compounds with potential cardiac liability at the earliest stages of drug development.

Among some 200 hereditary mutations in KCNH2 that cause type 2 long QT (LQT2) [4], about 67% are missense alterations, the majority of which cause trafficking defects; that is, an overall reduction of cell surface hERG protein [5]. In most cases, when these mutants are expressed in HEK293 cells, the trafficking defects can be 'rescued' or at least partially corrected by incubating cells at 27°C or by adding hERG blockers such as E4041, or the Ca^2+^-ATPase inhibitor thapsigargin [5-11]. Low temperature maintenance has also been reported to increase the surface expression of trafficking defective mutants of CFTR [12] in stably transfected polarized CFBE41o-cells, wild type α7-nicotinic acetylcholine receptor in SH-EP1 cells [13], as well as the production of a number of recombinant proteins that are soluble and secreted in CHO cells [14]. However, the mechanism of these temperature effects is not well understood.

Here we report an increase in expression of wild type hERG at lower growth temperatures and the characterization of the accumulation of hERG protein in intracellular vesicles in CHO-K1 cells. Our results demonstrate that low temperature growth can be an important tool in the development of robust drug screening assays for difficult-to-express ion channels such as hERG.

## Results

### Increase in intracellular and cell surface hERG protein expression levels when cells were maintained at 30°C

**Figure 1:**
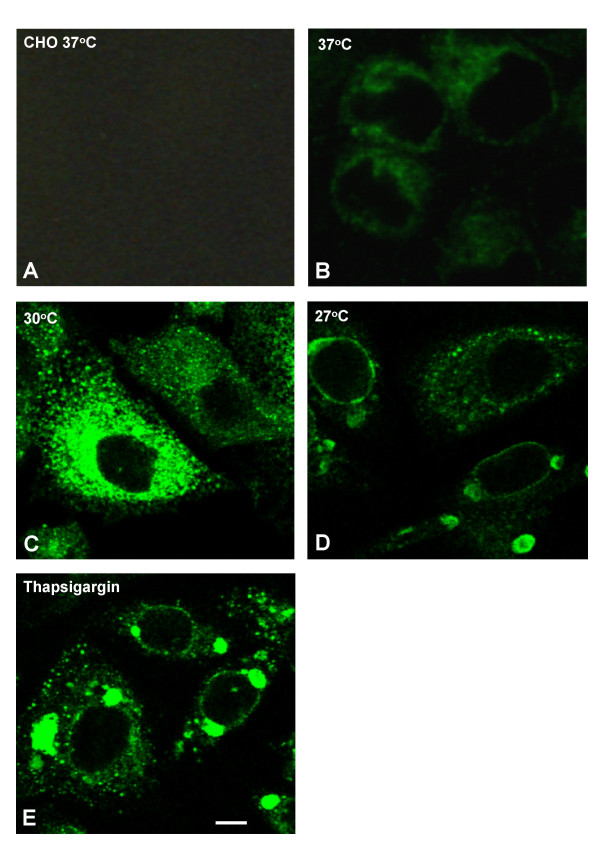
Subcellular localization of expressed hERG. Representative confocal microscopy images of cells fixed, permeablised and immunolabelled with anti-hERG antibody C20. CHO and CHO hERG cells grown at 37°C were split and kept subconfluent for 3 d at 37 (A,B), 30 (C), or 27°C (D). Additional samples were kept at 30°C for 1 d, followed by 24 h incubation at the same temperature with 1 μM thapsigargin (E). Bar, 5 μm.

A number of CHO-K1 stable cell lines over-expressing wild type hERG were established. Immunocytochemistry and confocal microscopy showed that most of the expressed hERG protein was located intracellularly and very little was associated with the cell surface (Fig. 1A–B). As low temperature cultivation has been reported to increase the surface expression of some membrane proteins, as well as the production of some non-membrane associated proteins [14,5,13], we looked at the effect of this treatment on wild type hERG expression.

**Figure 2:**
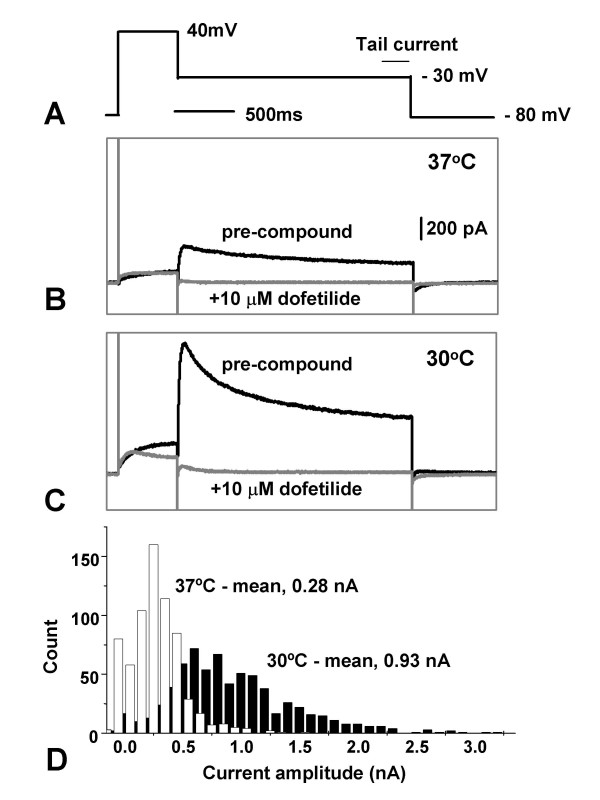
Increased functional expression of hERG at 30°C. HERG-expressing CHO cells were grown at 37°C (Materials and Methods), and then split and kept subconfluent at either 37 or 30°C for 3 d. Voltage command protocol (A). HERG currents were averaged from the last 200 mS of the -30 mV inactivation step before compound addition, subtracting the same recording after the application of 10 μM dofetilide. Patch clamp recordings made with an IonWorksHT instrument from a representative cell maintained at either 37 or 30°C (B,C, respectively). The black traces are recordings before compound addition. The grey traces are recordings following 10 μM dofetilide addition. It is noticeable that the difference at the initial phase of the -30 mV step was even bigger between the two temperatures. Population analysis of the hERG currents using three 384 well patch plates each (ie. >1000 recordings) for cells grown at 37 (open bars) and 30°C (black bars; D).

CHO hERG cells were normally maintained in culture at 37°C. Three days after switching to 30°C, hERG channel activity was measured by planar patch clamp electrophysiology using an IonWorksHT automated system. Current amplitudes measured at the tail end of a -30 mV inactivation step (see Fig. 2A, for voltage command protocol), were 3-fold higher following incubation at 30°C with a mean of 0.93 nA (*n *= 686), compared to 0.28 nA (*n *= 657, *p *< 0.0001) from the 37°C cells (Fig. 2B–D). The proportion of cells expressing tail currents above a threshold level of 0.1 nA (sufficient, for example, for high throughput compound screening) was also notably higher (96% vs 75%). These results suggest there is a general increase in expression of functional hERG protein on the cell surface of all cells examined at the lower temperature. An elevation of total channel protein associated with the cell membrane was observed at 30°C, as detected by Western blot (data not shown). Analysis by confocal microscopy confirmed these findings and also showed that, both at 37 and 30°C, hERG was largely concentrated in punctate cytoplasmic structures which became much more prominent at lower temperature (Fig. 1C, D).

Similar increased expression was observed with HEK293 cells expressing recombinant hERG (data not shown), as well as with other ion channel proteins, such as the nicotinic receptor subunits α4 and β2 (data not shown).

### Quantification of changes in intracellular and surface hERG protein expression by flow cytometry

**Figure 3:**
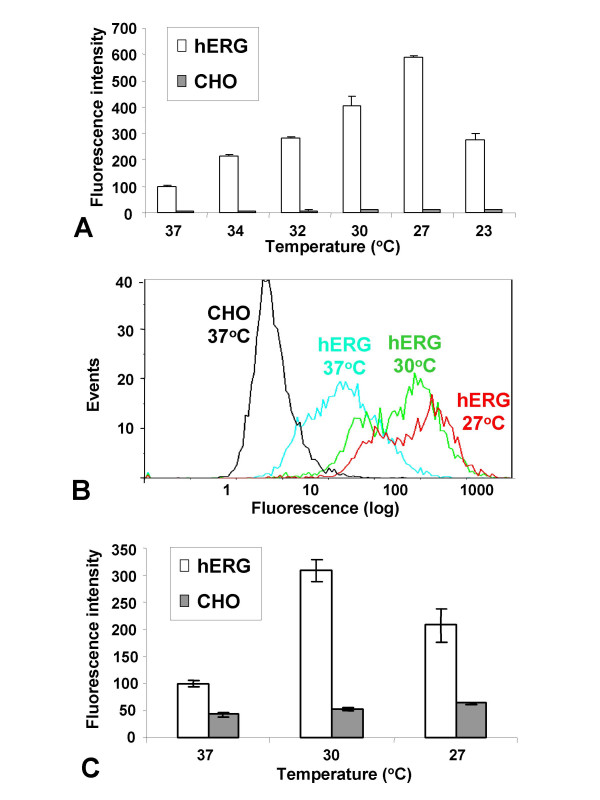
Quantification of total and surface-associated hERG protein in cells maintained at lower temperatures. Cells were cultured for 3 d and analyzed by flow cytometry. Data were averaged from 6 samples, each of 5000 cells (un-gated), and normalized against CHO hERG at 37°C. Normalized fluorescence intensity of fixed and permeablized CHO hERG (open bars) and untransfected control CHO cells (filled bars) at the respective temperatures stained with antibody C20 (which is raised against a C-terminal peptide sequence; A). Representative population fluorescence plots for CHO hERG cells from 37, 30 and 27°C in the same experiment as A (B). Normalized surface fluorescence intensity of non-fixed and non-permeablised CHO hERG (open bars) and control cells (filled bars) maintained at the respective temperatures stained with antibody 2110 (which is raised against a peptide between TM1 and TM2 which is predicted to be located on the surface of the plasma membrane; C).

Following the initial observations described above, flow cytometry was employed to quantify the increase in hERG expression occurring at lower temperatures. In the first experiment, total cellular hERG protein was measured using fixed and permeabilized cells labelled with an antibody (C20) raised against a peptide sequence located at the C-terminus of the protein which is predicted to be on the intracellular side of the plasma-membrane (or on the surface of internal membrane-bound organelles). Maintenance of the cells at 34, 32, 30, 27 and 23°C for 3 d increased total cellular hERG immunofluorescence compared with 37°C by some 2-, 3-, 4-, 6- and 3-fold, respectively. Thus, the total hERG protein expression peaked at 27°C, and then tailed off when the temperature was reduced further to 23°C (Fig. 3A, B). Extending the growth period from 3 to 7 d increased the expression still further, giving levels of ~6- and 10-fold higher than 37°C, at 30 and 27°C, respectively (data not shown), suggesting the effect was cumulative over time. Since nearly all of the immuno-fluorescence was cytoplasmic, these measurements largely reflect the amount of intracellular located hERG protein.

In a separate experiment, cell surface hERG was quantified by flow cytometry using cells that were not fixed or permeablized, and labelled with an antibody (2110) raised against a peptide between TM1 and TM2, which is predicted to be located on the surface of the plasma membrane. Following 3 d incubation, the increase in hERG fluorescence was ~3.5-fold at 30°C, and 2-fold at 27°C (Fig. 3C). These results suggest that the amplitude of increase of hERG on the cell surface was similar to that in the cytoplasm at 30°C, while at 27°C, a higher proportion of the protein remained intracellular.

### HERG electrophysiological activity was highest at 30°C, but ^3^H-dofetilide binding activity was optimal at 27°C

The flow cytometry experiments described above showed that the optimal cultivation temperatures for intracellular and surface hERG protein expression were different. We next compared the level of functional activity expressed at 30 and 27°C, using two types of assay, patch clamp electrophysiology and ^3^H-dofetilide binding. As before, the patch clamp experiments were carried out by automated planar array electrophysiology using an IonworksHT instrument which allows larger cell samples to be examined, thereby enabling greater statistical precision. In addition, unlike manual patch clamp, cells are sampled randomly from the total population, giving functional data that is more comparable to the protein expression data obtained by flow cytometry.

**Figure 4:**
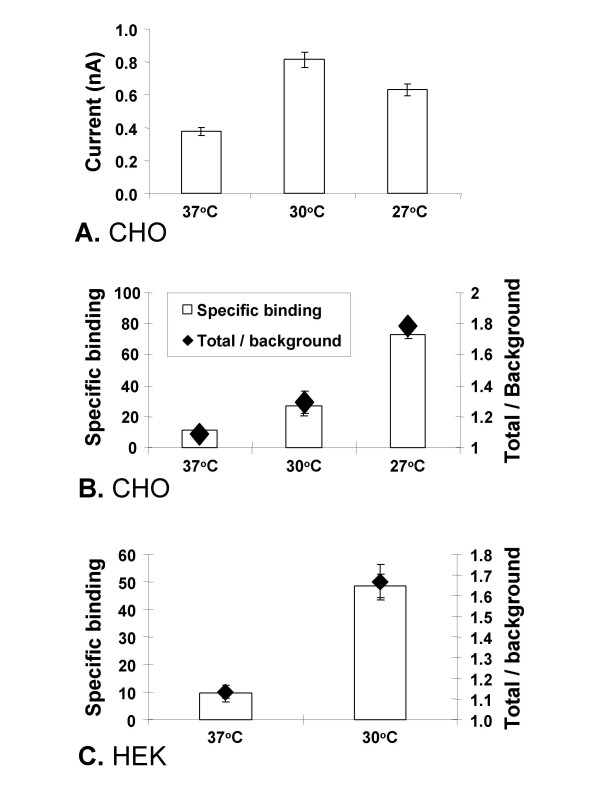
Comparison of hERG activity measured by patch clamp and ^3^H-dofetilide binding in cells maintained at different growth temperatures. CHO hERG cells grown at 37°C were split and kept subconfluent at 37, 30 or 27°C for 3 d (A,B). HEK293 hERG was split and kept at 37 or 30°C for 24 h (C). Mean tail currents recorded by patch clamping (IonWorksHT) from 4 independent experiments, 32–64 cells were patched for each data point in every experiment (A). ^3^H-dofetilide binding activity for CHO hERG and HEK hERG membrane preps respectively (B,C). Each data point was from 2 independent experiments, each of 3 repeats. Background (non-specific) binding was measured in the presence of an excess amount of non-radioactive dofetilide. Specific binding (open bars) was calculated as the total counts minus background counts and the total: background ratio (diamonds) was calculated as the total counts divided by the background counts.

HERG electrophysiological activity (Fig. 2A) was elevated at both 30 and 27°C compared to 37°C, and was highest at 30°C (Fig. 4A). The average currents at the tail end of the -30 mV inactivation step from cells at 37, 30 and 27°C were 0.38 (*n *= 133), 0.81 (*n *= 118) and 0.63 (*n *= 118) nA respectively, and are statistically different from each other (37/30°C *p *< 0.0001; 37/30°C *p *< 0.01, 30/27°C *p *< 0.05). On the other hand, although the level of specific ^3^H-dofetilide binding activity also increased at both temperatures, peak levels occurred at 27°C (Fig. 4B). Thus the ratios of total binding to non-specific binding from cells at 37, 30 and 27°C were 1.09, 1.29 and 1.78, respectively (37/30°C *p *< 0.05; 37/27°C *p *< 0.0001, 30/27°C *p *< 0.0001). Thus peak ion channel activity, which measures functional cell surface hERG, occurred when surface-expressed hERG protein levels were highest (Fig. 3C), whereas maximum binding activity, which measures ligand binding in total crude membrane extracts composed of both intracellular and plasmic membranes, was correlated with peak expression of total hERG protein (Fig. 3A, B). These results strongly suggest that although the bulk of the protein is trapped inside the cell, it is nevertheless expressed in a form capable of binding specific ligands.

The effect of lower growth temperature on hERG expression in HEK293 cells was then examined. Similar to CHO hERG cells, growing HEK293 hERG cells at 30°C also increased hERG current density measured by patch clamping (IonWorks, data not shown), and specific ^3^H-dofetilide binding (Fig. 4C). The ratio of total binding to non-specific binding from cells at 37 and 30°C were 1.13 and 1.67 respectively (*p *< 0.001).

### Accumulation of hERG-bound intracellular vesicles at lower temperature

Immunocytochemistry and confocal microscopy were carried out to further characterize the subcellular localization of hERG under different conditions. At 30°C or higher, hERG immunofluorescence was mostly located in vesicle-like punctate structures in the cytoplasm (Fig. 1B, C). At 27°C, in many cells, large vesicle/vacuole-like structures appeared in which much of hERG immunofluorescence was apparently located at their periphery (Fig. 1D). Similar hERG-loaded large/vacuole-like vesicles were rare in 30°C cells, but became abundant when combined with the presence of the endoplasmic reticulum Ca^2+^-ATPase inhibitor thapsigargin (Fig. 1E). Growth at 37°C in the presence of the compounds also led to the appearance of similar large/vacuole-like vesicles, albeit in a smaller population of cells (data not shown).

**Figure 5:**
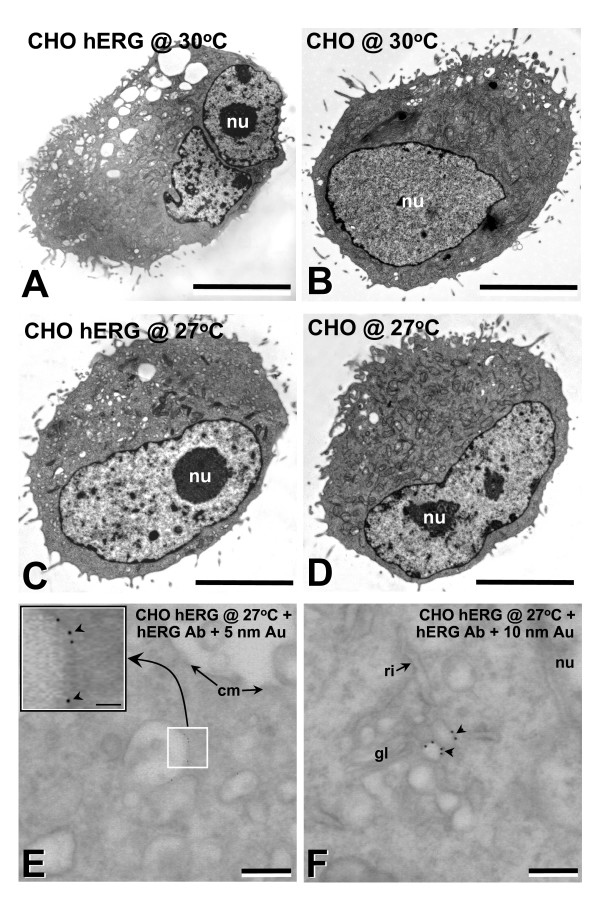
Ultrastructural characteristics of untransfected control and hERG transfected CHO cells. CHO hERG or untransfected cells from 37°C were split and kept subconfluent for 3 d at 30°C or 27°C. At 30°C, hERG transfected CHO cells showed numerous large vesicle/vacuole-like structures (A,B), which were fewer in number in untransfected 30°C CHO cells (B). At 27°C, the number of vesicle/vacuole-like structures were reduced compared to the 30°C cells (C,D *cf*/A,B). Immunolocalized hERG + 5 and 10 nm colloidal gold secondary antibody was present on vesicle/vacuole-type membrane (E,F, respectively). hERG + 10 nm colloidal gold secondary labelling was present adjacent to golgi (F). cm, cell membrane; gl, golgi; nu, nucleus. Bar, A-D,5 μm. E,F, 250 nm. E inset, 100 nm.

Ultrastructural studies showed a significant increase in the number and size of vesicles in hERG transfected cells at both 30 and 27°C (Fig. 5A, C), compared with untransfected cells (Fig. 5B, D). High resolution immunogold labelling showed that hERG was specifically associated with the surface membrane of these structures (Fig. 5E, F).

Endocytic trafficking of membrane vesicles can be affected by the microtubule-disrupting agent nocodazole [15-18]. As hERG is found to be accumulated in intracellular vesicles, the effect of the compound on hERG expression was investigated. Incubation of cells with 25 μM of nocodazole at 37°C for 24 h led to a 60% increase in the amount of total cellular hERG protein measured by flow cytometry, and a 90% increase of hERG currents determined by patch clamping (IonWorks). Expression of hERG increased significantly at 30°C, but did not increase further in the presence of nocodazole (data not shown). These results suggest that nocodazole can be a valuable tool in boosting hERG activity at 37°C, but not at the lower temperature.

## Discussion

Stable over-expression of many recombinant membrane associated proteins can be problematic, and overloading the cells with such proteins can often be cytotoxic (for review, [19]). In these cases, the protein often remains primarily intracellular and can be rapidly degraded. The lack of significant quantities on the cell surface is often a major hindrance for drug discovery since it renders the development of robust assays that can be used for compound screening highly problematic. Low temperature maintenance has been reported to increase the yield of some recombinant proteins, including those that are soluble, secreted [14], and membrane-bound [13]. In this study, using a variety of techniques including flow cytometry, immunoblotting and confocal and high resolution immunocytochemistry, we have demonstrated a cumulative increase over time in the level of cellular and surface hERG protein when expressing cells are cultured at lower temperature. These findings may be general since they occur in cell backgrounds other than CHO, including HEK293, and for some other channel proteins such as nicotinic acetylcholine receptor subunits ([20,13] and data not shown).

Confocal and high resolution ultrastructural immunocytochemistry showed that hERG protein expression was most prominent in intracellular vesicle/vacuole-like compartments. These became more abundant at 30°C and much larger when incubated at 27°C or when treated with the ER Ca^2+^-ATPase inhibitor thapsigargin. Both confocal and electron microscopy detected hERG protein predominantly on the peripheral membrane of these vesicles. It is conceivable that such accumulation and enlargement of the vesicle/vacuole-like structures could be the result of a blockade en route to their destruction. Interestingly, similar to low temperature, thapsigargin can also "rescue" trafficking defective hERG mutants [5,6]. Our results for the first time suggest a possible common mechanism for these two types of treatments.

When over-expressed, some membrane proteins are readily endocytosed after reaching the cell surface; some are destined for degradation in lysosomes while others can be recycled back to the cell surface [21-23]. The surface expression of hERG in HEK293 cells is strictly regulated, internalisation and lysosomal degradation can be rapidly induced by ceramide [24]. In Swiss 3T3 cells, uptake of exogenous materials and transport through the endocytic pathway to lysosomes slowed at lower temperature [25]. In HeLa cells, 20°C caused a small reduction of endocytosis, but a near complete block of the removal of internalised dextran-containing late endosome vesicles [15]. In rat hepatocytes, temperatures of 16–20°C blocked the transition from early to late endosomes, and from late endosomes to lysosomes [26,27]. Uptake and catabolism of endocytic particles continued, but progressively slowed as the temperature decreased from 35 to 20°C. Fusion of the vesicles to lysosomes did not occur at 20°C or below, while endocytosis and internal vesicle movement continued and only stopped at a much lower temperature, below 10°C [28]. Our results demonstrating the accumulation of intracellular vesicles loaded with hERG channel protein that is apparently fully able to bind^3^H-dofetilide, together with an increased stability of the protein at the lower temperature (detected by pulse chase labelling, data not shown), are in clear agreement with these previous reports and are consistent with a model of reduced trafficking of vesicles further down the endocytic pathway for degradation, albeit perhaps in combination with a small, but significant reduction in endocytosis.

The above data are further strengthened by the present results with nocodazole. The compound has no effect on endocytosis, but blocks microtubule-dependant steps later in the endocytic pathway. In HeLa cells, nocodazole prevented transferrin transport from early endosomes to the peri-nuclear recycling compartment [15], and caused accumulation of endosomal carrier vesicles, a compartment intermediate between early and late endosomes [16]. In a similar manner in BHK cells, nocodazole caused the accumulation of spherical vesicles that has little fusion activity, suggesting that transport between early and late endosomes requires intact microtubules [17]. Furthermore, pre-treatment of CHO, 3T3, HeLa, and NRK cells with nocodazole inhibited the content exchange of lysosomes in cell fusion experiments [18]. In our experiments, the effects of nocodazole and low temperature were similar, but not additive. This is consistent with the effect of nocodazole being due to blockade of endocytic trafficking rather than cell cycle progression, since in the latter scenario the increase in hERG expression would have been expected to be larger when combined with 30°C. The cells can be maintained at 30°C continuously with healthy exponential growth, albeit at a slower rate, without any sign of cell cycle arrest found in the cells treated with nocodazole, such as complete growth arrest and multi-nucleation. The accumulation of hERG at 30°C was greater than in the presence of nocodazole even at 37°C, suggesting that cell cycle arrest alone can not explain this temperature effect.

It is conceivable that a number of other mechanisms could also, perhaps to a lesser extent, contribute to the temperature effect on hERG expression, such as an improvement in protein folding, maturation or trafficking to the cell surface. While a number of hereditary mutations in hERG render the protein misfolded and destined for ER-associated degradation through proteosomes, there is little evidence of this happening for the wild type protein [6,9,29,11,30].

## Conclusion

Our results demonstrate that low temperature maintenance can be used to boost the functional expression of some difficult-to-express cell surface membrane associated proteins such as the hERG channel, and significantly improve the quality of compound screening assays ranging from patch clamping to radio-ligand binding. The data also sheds some light on the mechanism of this temperature effect.

We hypothesis that at the normal culturing temperature of 37°C, some of the over-expressed hERG channel protein reaches the cell surface, but is rapidly internalized. This and perhaps other exit trafficking routes sends the protein to intracellular vesicles destined for degradation. Transfer into lysosomes and, to a lesser extent, endocytosis are disproportionately slowed as the temperature is reduced leading to the accumulation of hERG-loaded vesicles as well as an accumulation of hERG protein on the cell surface. Some of the retained hERG protein can perhaps be trafficked back to the cell surface due to constitutive endocytic recycling, similar to that occurs with some other membrane proteins [22,31]. The aggregation of these effects leads to an increase in protein retention both inside the cell and on the surface membrane. It is likely that an increase in cellular retention of trafficking defective hereditary mutant hERG proteins, through this mechanism, could contribute to the low temperature channel "rescue", in addition to improvements perhaps in folding and trafficking through the ER-Golgi network [6,9,29,11].

## Methods

### Materials

All cell culture reagents were obtained from Invitrogen unless otherwise stated. Goat anti-hERG intracellular peptide antibody C20 and rabbit anti-nicotinic acetylcholine receptor α4 and β2 antibodies were obtained from Santa Cruz. Anti-hERG extracellular peptide (AFLLKETEEGPPATEC) antibody 2110 was raised in rabbit and affinity purified. Alexa488 secondary antibodies were obtained from Cambridge Bioscience. Nocodazole was supplied by Sigma.

### Stable cell line generation and maintenance

Wild type hERG (cloned from cardiac cDNA, predicted peptide sequence identical to Q12809 in SWISSPROT database) stable cell line was generated in CHO-K1 (ATCC N° CRL 9618) and HEK293 cells, and nicotinic acetylcholine receptor α4β2 stable cell line was generated in HEK293 cells, in all cases using IRES expression constructs [32]. Purified plasmid DNA (1 μg) was used to transfect ~5 × 10^6 ^cells by electroporation (standard protocol with BIORAD Gene Pulser II). After 24 h growth, chemical selection was applied by addition of 500 μg ml^-1 ^geneticin for hERG, or 200 μg ml^-1 ^hygromycin plus 500 μg ml^-1 ^geneticin for nicotinic acetylcholine receptor α4β2. After a further 2 wks in culture, antibiotic-resistant clones were isolated, expanded under antibiotic selection and subsequently stored in liquid nitrogen until subsequent analysis. Cells were maintained in α-MEM medium with glutamax containing a 1% solution of non-essential amino acids and 10% FBS for clone selection, and in DMEM Ham's F12 medium containing glutamax and 10% FBS for the subsequent experiments, unless stated otherwise.

### Immunofluorescence confocal microscopy

Cells were fixed with Parafix (Pioneer Research Chemicals), quenched in 15 mM glycine for 30 min, and permeabilized with 0.1% saponin in PBS plus 1% BSA. Cells were then incubated with primary antibody, followed by secondary antibody in permeabilization buffer, for 1 h each, with washing between incubations. After extensive washing over a 45 min period, cells were mounted in Citifluor (Agar Scientific) on glass slides and examined on a Leica TCS-4D confocal microscope (Leica) using a 40× objective oil lens.

### Flow cytometry

For measuring total cellular protein, cells were removed from adherent culture and fixed with Parafix for 10 min. Following a PBS wash, cells were resuspended in FC buffer (PBS plus 1 mM EDTA, 0.1% BSA, 0.5% Triton ×-100, and 0.02% sodium azide), and incubated with a mixture of 1/500 dilution of primary and secondary antibodies for 1 h at room temperature. Following one wash, the cells were resuspended in PBS and analyzed on a FC500 flow cytometer (Beckman Coulter). The protocol for staining non-permeablized cells was nearly identical except that the cells were not fixed, and Triton ×-100 was omitted from the FC buffer.

### IonWorksHT patch-clamp electrophysiology recording

Planar array patch clamp electrophysiological recordings were carried out using a 384-well IonWorksHT reader (Molecular Devices [33]). A fresh cell suspension in external PBS was placed in the cell boat on the deck of the instrument and the experiment started within 2 min. Following a 10 min period for the cells to seal to the substrate, the amphoterocin (100 μM) containing internal solution (140 mM KCl, 1 mM MgCl_2_, 1 mM EGTA, 20 mM HEPES, pH 7.3 with KOH) was introduced and left to equilibrate for a further 4 min. During this time the perforated patch clamp configuration was achieved with estimated access resistances of 5–10 MΩ. Cells were clamped at a holding potential of -80 mV for 30s before ionic currents were measured using the following step protocol: 40 mV 500 ms, -30 mV 2000 ms before returning to -80 mV (Fig. 2A). The same voltage step was applied after the addition of 10 μM dofetilide. HERG currents were collected at the tail end of the -30 mV inactivation step, by averaging recordings from the last 200 ms and subtracting the same current after compound addition. Currents greater than 0.1 nA after subtraction were included in the analysis, and commonly represent around 80% of all cells recorded from. Statistics was carried out using unpaired students t-test, and a *p *< 0.05.

### ^3^H-dofetilide binding assay

Cells were removed from the bottom of tissue culture vessels by 5 min incubation in Versene (Invitrogen), diluted with 10 times the amount of growth medium, and pelleted by centrifugation. Each 0.1 ml aliquot of the pellet was resuspended in 1 ml of lysis buffer (50 mM HEPES, pH 7.4, 1 mM EDTA, 1 tablet complete protease inhibitor cocktail per 50 ml) and passed 22 times through a 25 G fine needle with a 1 ml disposable syringe. This was followed by centrifugation at 1,500 rpm for 10 min at 4°C in a bench-top centrifuge to clear cell debris, and a further spin of 14,000 rpm at 4°C to pellet the membrane. The pellet was resuspended in 200 μl of lysis buffer without protease inhibitors, snap frozen on dry ice and stored at -80°C.

Binding assays were performed in 384-well format using 120 μg per well wheat germ agglutinin SPA beads (Amersham) pre-coated with 2 μg membrane protein, and 5 nM^3^H-dofetilide (Amersham), in buffer containing 25 mM HEPES, 1.2 mM MgCl_2 _(adjusted to pH 7.4 with KCl), and 0.2% pluronic. Following 0.5 h incubation, plates were read on a ViewLux instrument (Perkin Elmer 1430).

### Electron microscopy

For conventional electron microscopy, cell pellets were fixed in 3% fresh glutaraldehyde in PBS, pH 7.4, and processed using standard procedures [34]. Cells were photographed at × 2,500–60,000 in a Phillips 7100 TEM.

For high resolution immunolocalization, cell pellets were frozen in liquid nitrogen at ~2100 bar (BAL TEC, HPM 010), freeze-substituted (Leica, AFS) at -90°C in 0.1% uranyl acetate in acetone over 4 d and embedded in LR gold polymerized under UV light at -25°C. Thin sections were cut and placed on formvar and carbon coated nickel slot grids and sequentially incubated in PBS containing 1% normal donkey serum and 0.2% Tween-20 (blocking buffer; 30 min), and antibody raised against human HERG (1:100; Santa Cruz; sc-15968) in blocking buffer for 18 h at 4°C, then rinsed and incubated in secondary antibodies conjugated to 5 and 10 nm colloidal gold (1:40; British Biocell) in 0.01% Tween-20. Sections were subsequently treated with 1% glutaraldehyde in PBS, and stained conventionally.

## Authors' contributions

MC conceived of the study, was the primary designer, coordinator and executer and drafted the manuscript. SS carried out the EM studies and helped to draft the manuscript. VD conducted some of the work on expression and characterization. YC carried out the work on nicotinic acetylcholine receptor. HH conducted some of the flow cytometry experiments. BH helped with methods for flow cytometry and membrane preparation. HM and DT carried out and helped with IonWorks experiments. JC helped with the design and coordination of the experiments and the drafting of the manuscript.
